# Long-Chain Omega-3 Fatty Acids Supplementation Accelerates Nerve Regeneration and Prevents Neuropathic Pain Behavior in Mice

**DOI:** 10.3389/fphar.2017.00723

**Published:** 2017-10-17

**Authors:** Rafaela V. Silva, Julia T. Oliveira, Bruna L. R. Santos, Fabiana C. Dias, Ana M. B. Martinez, Cleverton K. F. Lima, Ana L. P. Miranda

**Affiliations:** ^1^Laboratório de Estudos em Farmacologia Experimental, Departamento de Biotecnologia Farmacêutica, Faculdade de Farmácia, Universidade Federal do Rio de Janeiro, Rio de Janeiro, Brazil; ^2^Laboratório de Neurodegeneração e Reparo, Departamento de Patologia, Faculdade de Medicina, Universidade Federal do Rio de Janeiro, Rio de Janeiro, Brazil

**Keywords:** omega-3 PUFA, neuropathic pain, fish oil, nerve regeneration, peripheral nerve injury, docosahexaenoic acid

## Abstract

Fish oil (FO) is the main source of long chain omega-3 polyunsaturated fatty acids (ω-3 PUFAs), which display relevant analgesic and anti-inflammatory properties. Peripheral nerve injury is driven by degeneration, neuroinflammation, and neuronal plasticity which results in neuropathic pain (NP) symptoms such as allodynia and hyperalgesia. We tested the preventive effect of an EPA/DHA-concentrate fish oil (CFO) on NP development and regenerative features. Swiss mice received daily oral treatment with CFO 4.6 or 2.3 g/kg for 10 days after NP was induced by partial sciatic nerve ligation. Mechanical allodynia and thermal hypernociception were assessed 5 days after injury. CFO 2.3 g/kg significantly prevented mechanical and thermal sensitization, reduced TNF levels in the spinal cord, sciatic MPO activity, and ATF-3 expression on DRG cells. CFO improved Sciatic Functional Index (SFI) as well as electrophysiological recordings, corroborating the increased GAP43 expression and total number of myelinated fibers observed in sciatic nerve. No locomotor activity impairment was observed in CFO treated groups. These results point to the regenerative and possibly protective properties of a combined EPA and DHA oral administration after peripheral nerve injury, as well as its anti-neuroinflammatory activity, evidencing ω-3 PUFAs promising therapeutic outcomes for NP treatment.

## Introduction

Omega-3 polyunsaturated fatty acids (ω-3 PUFAs) are long chain fatty acids (18–22 carbons) with the first double bond at the carbon number 3. They are well known for their health benefits in cardiovascular protection and as anti-inflammatory (Calder and Yaqoob, 2009). Eicosapentaenoic acid (EPA) and docosahexaenoic acid (DHA) are the main long chain ω-3 PUFAs studied and have demonstrated efficacy as pain modulators, reducing pain symptoms in patients with osteoarthritis, rheumatoid arthritis, and inflammatory bowel disease (Ruggiero et al., 2009; Hill et al., 2016). Because humans are unable to synthesize these elongated PUFAs and can only poorly convert α-linolenic acid into EPA and DHA, these fatty acids are generally obtained through consumption of seafood, especially oily fish (Calder and Yaqoob, 2009).

Fish oil (FO), which is the main source of EPA and DHA, have exhibited analgesic and anti-inflammatory activities in animal models of chronic pain (Veigas et al., 2011; Lobo et al., 2016). The exact mechanisms underlying the effect of ω-3 PUFAs on reducing pain sensitivity is not fully elucidated (Trépanier et al., 2016). However, animal studies have attributed PUFAs benefits to modulation of neuronal activity, regulation of immune cells response and also production of more potent pro-resolving and anti-inflammatory lipid mediators, such as resolvins (Lu et al., 2013; Heng et al., 2015; Lobo et al., 2016).

Neuropathic pain (NP) is a debilitating heterogeneous disorder estimated to be prevalent in up to 10% in the general population (van Hecke et al., 2014). Peripheral NP emerges from injury or malfunction of somatossensory system generally caused by direct trauma, disease, virus infection, or neurotoxic chemicals affecting somatosensory system (Treede et al., 2008; Baron et al., 2010).

Following nerve injury, axon degeneration occurs within 24 h with myelin sheath disintegration (Gaudet et al., 2011). Meanwhile, a critical neuroinflammatory process is initiated with immune cells being activated and recruited into peripheral and central nervous system, in addition to activation of astrocytes and microglia, immune-like cells of the CNS (Ellis and Bennett, 2013). Together, these cells are responsible for secreting a number of inflammatory mediators including cytokines, such as TNF, chemokines and lipid mediators essential for neuronal sensitization (Ignatowski et al., 1999; Ellis and Bennett, 2013). As a result of a maladaptative response, central and peripheral sensitizations are developed, producing hypersensitivity symptoms, including allodynia and hyperalgesia as well as ongoing spontaneous pain (Costigan et al., 2009).

An increasing number of researches have aimed to address new targets for NP treatment as current pharmacological treatment is based on neuronal modulation and is only partially effective, whereas classical anti-inflammatory drugs alone are inefficacious (Dworkin et al., 2007). In NP associated to peripheral nerve injury, promotion of nerve regeneration by a combination of vitamin B, nucleotides and folic acid has been employed to improve nerve regeneration and NP symptoms (Negrão et al., 2014). Due to multiple mechanisms involved in NP pathophysiology and its complex pharmacological management, prevention of NP development following nerve injury seems to be a reasonable approach to avoid maladaptive response and its consequences. The benefits of concentrate fish oil (CFO) (84% of EPA and DHA ethyl esters) in peripheral NP have not been investigated yet, neither its ability to prevent NP development. In a previous study from our group CFO displayed superior inhibition of inflammatory pain when compared to FO in rodents because of its increased composition in EPA and DHA (Lobo et al., 2016). In this context, this study aimed to evaluate the effect of CFO oral treatment in preventing NP development after partial sciatic nerve ligation (PSNL) in mice, shedding light on its mechanism of action.

## Materials and Methods

### Animals

Adult female and male Swiss mice from the breeding unit of the Faculty of Pharmacy (UFRJ, Brazil) weighing from 20 to 25 g were used in this study. All animal protocols were previously approved by the Ethical Animal Care Committee of Federal University of Rio de Janeiro (CEUA-UFRJ) under the number 011/16, in compliance with international guidelines (EU Directive 2010/63/E). Animals were housed under controlled conditions of temperature at 22°C ± 1°C, with light/dark cycle of 12 h and with food and water *d libitum*. Animals were randomly distributed into experimental groups. Each experimental group contained equal amount of male and female mice.

### Partial Sciatic Nerve Ligation

Neuropathic pain was induced by PSNL as previously described (Malmberg and Basbaum, 1998). Briefly, anesthetized animals with ketamine 100 mg/kg + xylazine 10 mg/kg (i.p.) underwent a surgical procedure to allow ligation of 1/3 to 1/2 of left sciatic nerve at the mid thigh level with 4-0 Catgut suture. At the end, the skin was sutured with a 5-0 nylon suture thread. Sham operated mice had their sciatic nerve only exposed but not ligated. On the 9th day after surgery, animals were euthanized and tissue samples collected for further analysis.

### Treatment Protocols

Concentrate fish oil, containing 4.6% EPA and 3.6% DHA, was obtained commercially (OMACOR^®^, Pierre Fabre). Based on previous studies, two doses were selected 2.3 g/kg and 4.6 g/kg of EPA content, respectively 1.9 and 3.8 g of DHA/kg (Lobo et al., 2016). Two groups of animals received daily oral treatment, one for 10 days starting at the day of the surgery (preventive protocol) and another for 5 days starting 5 days after nerve surgery (therapeutic protocol). Gabapentin, a NP first-line treatment drug, was orally administered at the dose of 100 mg/kg. Vehicle group received Arabic gum 5% (5 μL/g). All oral treatments were performed by gavage.

### Behavioral Hypersensitivity Tests

Mechanical allodynia was assessed using von Frey filaments from 0.008 to 2 g (Chaplan et al., 1994). Three days prior to behavioral testing, mice were placed on a grid platform inside acrylic boxes for up to 1 h a day. Basal withdrawal threshold was evaluated before the surgery and allodynia was assessed on 5th, 7th, and 9th days after surgery. Withdrawal threshold was determined as correspondent in grams of the thinnest filament eliciting a nociceptive response in mice. Measures are expressed as percentage of basal response, considered 100%.

Thermal hypernociception was assessed by Hargreaves test (Hargreaves et al., 1988). Three days prior to behavioral tests, mice were placed on the platform up to 30 min a day. Latency time for paw withdrawal was recorded from the moment of incidence of a beam of radiant heat (Ugo Basile) on left plantar surface to a nociceptive withdrawal response. Basal withdrawal latency time was measured before the surgery and hyperalgesia was assessed on 5th, 7th, and 9th days after surgery. Results are expressed as delta (Δ) of latency in seconds, which represent the difference between latency time for each time point and basal recordings.

### Open Field Test

The open field test was used to assess spontaneous locomotor activity after oral treatments. After 1 or 10 repeated administrations, mice were placed on the center of a 50 × 50 × 50 cm box/apparatus with a white floor divided in 9 equal squares. The number of squares crossed and rears were counted for 5 min.

### TNF Quantification

For quantification of TNF cytokine, spinal cord samples from L4-L6 section were homogenized with PBS containing protein inhibitor cocktail (1:100, Sigma). In sequence, samples were centrifuged at 10,000 rpm for 10 min at 4°C. The supernatant was collected and TNF quantified using an enzyme-linked immunosorbant assay kit (R&D Systems). Total protein was determined by the bicinchoninic acid (BCA) assay (Sigma). Results were expressed as relative TNF concentration in pg per mg of protein.

### Myeloperoxidase Activity Assay

Myeloperoxidase (MPO) activity was assessed as an indirect measure of neutrophil migration into sciatic nerve, as described previously (Cunha et al., 2008). Succinctly, tissue was homogenized in 200 μL hexadecyl trimethylammonium 0.5% buffer (HTAB) with protein inhibitor cocktail 1:100 and centrifuged at 13,200 rpm for 3 min. The supernatant was collected and the assay was performed on a 96-well plate. Each well received 100 μL of sample and 100 μL of a developing solution containing *O*-dianisidine dyhydrochloride 0.5 mM and 0.015% hydrogen peroxide. Absorbance was measured at 450 nm. Results are expressed in optical density (OD) per milligram of total tissue protein.

### Western Blot Analysis

DRG or sciatic samples were homogenized in lysis buffer (1% Triton, 20 mM Tris pH 7.6, 1 mM EDTA, 150 mM NaCl, 1 mM NaF, 1 mM NaVO3) with protein inhibitor cocktail (Sigma) followed by total protein quantification. Proteins were resolved in 12% SDS-PAGE gel and transferred to a nitrocellulose membrane (Bio-Rad). The membrane was then blocked with 5% non-fat milk in TBS-T solution for 1 h, followed by incubation with anti-GAP-43 (sciatic sample, Santa Cruz, RRID:AB_640874), anti-ATF-3 (DRG sample, Santa Cruz, RRID:AB_2258513) or anti-β-actin/actin primary antibody overnight at 4°C (1:1000, Santa Cruz, RRID:AB_2223228 /Millipore, RRID:AB_2223041). On the next day, anti-rabbit secondary antibody was incubated for 1 h and chemiluminescence was measured using a ChemiDoc XRS+ imaging system (Bio-Rad) after development in chemiluminescence reagent (Westar Nova 2011, CYANAGEN). Bands intensity was determined using ImageJ software (NIH, United States).

### Sciatic Functional Index (SFI)

Motor function was evaluated by the SFI test, based on a protocol previously described (Inserra et al., 1998). The animals’ paw prints were registered before surgery and weekly after surgery. Two measurements were taken from the prints: the print length (PL), corresponding to the distance from the heel to the third toe, and the toe spread (TS), corresponding to the distance from the first to the fifth toe. These measurements were taken from both injured (E, from experimental) and uninjured (N, from normal) paws and the SFI was calculated according to the following equation:

$$SFI=118.9\left(\frac{ETS-NTS}{NTS}\right)-51.2\left(\frac{EPL-NPL}{NPL}\right)-7.5$$

A *SFI* around zero corresponds to normal nerve function while a SFI around -100 represents total loss of sciatic nerve function.

### Light Microscopy

Two weeks after surgery, the animals were anesthetized (i.p.) with ketamine (100 mg/kg) and xylazine (15 mg/kg) and euthanized by intracardiac perfusion with a fixative solution (4% paraformaldehyde in 0.1 M phosphate buffer, pH 7.4). After perfusion, a segment of 2 mm of the sciatic nerve distal to injury site was harvested and immediately fixed by immersion in 2.5% glutaraldehyde overnight, washed in 0.1M cacodylate buffer (pH 7.4), and postfixed for 90 min. in 1% osmium tetroxide containing 0.8% potassium ferrocyanide and 5 mM calcium chloride in 0.1 M cacodylate buffer (pH 7.4). The segments were then washed in 0.1 M cacodylate buffer (pH 7.4) and stained in 1% uranyl acetate overnight. Nerves were dehydrated in graded acetone (30, 50, 70, 80, 90, and 100%), infiltrated with Embed-812 resin (Electron Microscopy Sciences) and polymerized at 600°C for 48 h. Semi-thin (500 ηm) cross-sections were obtained on an RMC MT-6000 ultramicrotome. The semi-thin sections were stained with 1% Toluidine Blue and examined under a light microscope (Zeiss Axioskop 2 Plus).

### Morphometric Assessment

Images of the semi-thin cross sections were captured under the light microscope at a 40x magnification and used for quantitative analysis. The total number of myelinated nerve fibers in each regenerated nerve was counted using ImageJ Software (1.42q, United States). A single-blind analysis was performed for this assessment.

### *In Vivo* Electrophysiological Recordings

For electrophysiological recordings, at the end of the second week of treatment animals were anesthetized and the left sciatic nerve was re-exposed. Recording electrodes were placed into the tendon and in the belly of gastrocnemius muscle and a stimulating electrode was placed at the proximal trunk of sciatic nerve. The ground electrode was inserted subcutaneously in the dorsal area. Compound muscle action potentials (MAP) were recorded at room temperature following application of electrical impulses (10 V, 0.01 s) using PowerLab data acquisition system and an Animal Bio Amp amplifier (AD Instruments). Amplitude, latency, and area of MAP registered were measured using LabChart data analysis software (AD Instruments). For each animal the results were expressed as mean of 3 recordings.

### Statistical Analysis

All data are presented as mean ± standard error of the mean (SEM). Data were statistically analyzed by the *t* Student’s test, One-way and Two-way ANOVA (Bonferroni’s and Dunnett’s Multiple Comparison post-test) for a significance level of *p* < 0.05. The analyses were performed using *GraphPad Prism*^®^ software v. 5.0.

## Results

### CFO Prevents Mechanical and Thermal Hypersensitivity Development after PSNL

**FIGURE 1:**
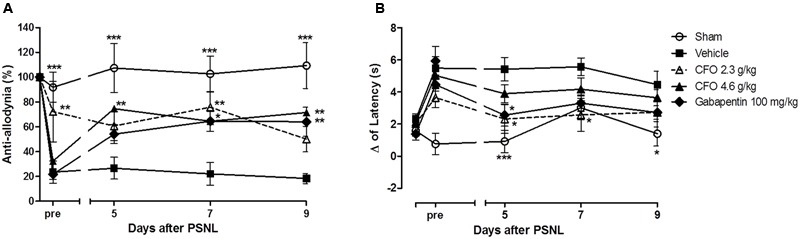
Effect of CFO on mechanical allodynia and thermal hypernociception after PSNL in preventive protocol. Percentage of basal mechanical withdrawal threshold represented as anti-allodynia **(A)**, *n* = 9–11 and delta (Δ) of latency time **(B)**, *n* = 6–9, before (pre) and 3 h after daily oral treatment. Two-way ANOVA followed by Bonferroni *post hoc* test, ^∗^*p* < 0.05, ^∗∗^*p* < 0.01, ^∗∗∗^*p* < 0.001 compared to vehicle group.

To examine the effect of ω-3 PUFAs on NP development, daily oral administration of CFO (2.3 or 4.6 g/kg) was initiated on the day of PSNL induction and repeated during 10 consecutive days. On the 5^th^ day post injury, thermal hypernociception and mechanical allodynia were assessed prior to treatment. CFO at 2.3 g/kg significantly prevented mechanical allodynia and reduced thermal hypernociception in mice compared to animals receiving vehicle only (**Figures 1A,B**). At this point, CFO 2.3 g/kg treated animals have consistent higher withdrawal threshold than vehicle and gabapentin groups, presenting 70% of anti-allodynic response. In addition, 3 h after oral administration, CFO 2.3 g/Kg remained as effective as gabapentin (100 mg/Kg) improving mechanical allodynia. The higher CFO dosage also diminished allodynic behavior after 3 h on days 5, 7, and 9 (**Figure 1A**).

Similarly, the delta of latency of CFO 2.3 g/kg group on the 5th day prior to treatment was 3.6 s, 1.6 s lower than the vehicle group (**Figure 1B**). At the same time, variation of latency of sham group was 0.7 s. In the following assessments, the latency time variation for CFO 2.3 g/kg group was equivalent to sham operated animals.

### Locomotor Activity Was Not Altered by CFO

**FIGURE 2:**
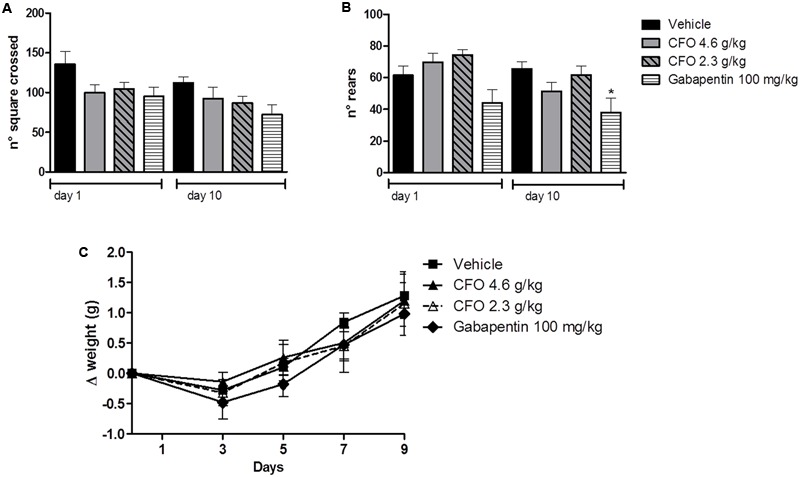
Effect of CFO on locomotor activity and body weight. Locomotor activity was evaluated in the open field test after 1 and 10 oral administrations in healthy animals, expressed as total number of squares crossed **(A)** and number of rears **(B)**. Body weight variation (Δ) was also assessed in these animals **(C)**. *n* = 7–8; One-way ANOVA followed by Bonferroni *post hoc* test, ^∗^*p* < 0.05 compared to vehicle.

The open field test has been used to predict the influence of drugs on motor activity. Thus, total number of squares crossed as an indication of the distance moved and total number of rears as an exploratory behavior were assessed for 5 min. Because most experiments were conducted in animals receiving daily treatment, locomotor activity of healthy animals receiving vehicle, CFO or gabapentin was evaluated on the first and last day of treatment. The number of squares crossed was not significantly different among groups, suggesting no alteration in locomotor capacity (**Figure 2A**). On 10th day, locomotor activity remained unaltered among groups (**Figure 2A**). Also, CFO did not alter the total number of rears after 1 or 10 days of treatment in comparison to vehicle (**Figure 2B**). However, gabapentin at 100 g/kg caused a change in the explorative behavior, statistically reduced on 10th day (**Figure 2B**).

Additionally, as an overall indication of well-being, body weight variation was calculated for the 10 days of treatment. No treatment induced a substantial change in weight gain, indicated as delta of weight, (**Figure 2C**).

### CFO Attenuates Neuroinflammation by Reducing Spinal Cord TNF Levels and Sciatic MPO Activity

**FIGURE 3:**
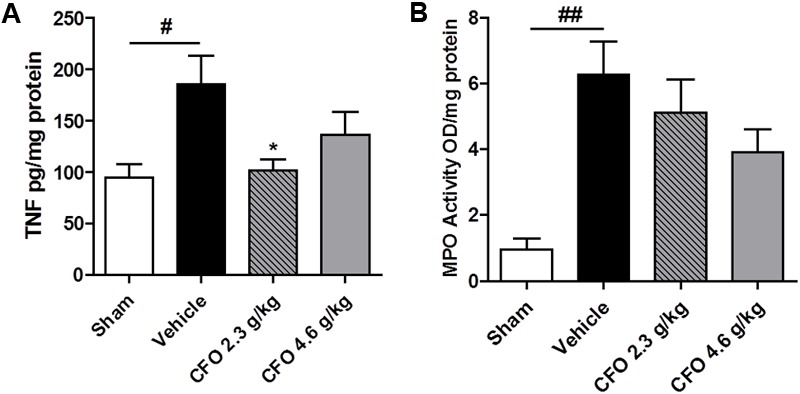
TNF and MPO levels after PSNL. Spinal cord TNF production **(A)**, *n* = 6–7. Sciatic nerve MPO activity **(B)**, *n* = 4. One-way ANOVA followed by Dunnett’s *post hoc* test, ^∗^*p* < 0.05 compared to vehicle group, ^#^*p* < 0.05, ^##^*p* < 0.01 compared to sham group.

Considering the contribution of neuroinflammation to the onset of peripheral NP, biomarkers of this process were analyzed. TNF, a key inflammatory cytokine required for peripheral and central sensitization, was quantified. Therefore, levels of TNF in the spinal cord 9 days after nerve injury were quantified. Opposed to the significant increase in TNF amount in spinal cord of vehicle treated animals, CFO 2.3 g/kg supplementation preserved TNF status at levels comparable to sham group (**Figure 3A**).

On the site of injury, neutrophils are the first immune cell to migrate and initiate the inflammatory response and its inhibition prevents hypersensitivity. MPO activity was used as an indirect marker of neutrophil migration into injured sciatic nerve. At the 9th, MPO activity was found to be highly elevated on sciatic nerve of neuropathic animals and both CFO doses administered caused a moderate reduction with no statistical difference (**Figure 3B**).

### Neuronal Damage after Nerve Injury Is Reduced by CFO Treatment

**FIGURE 4:**
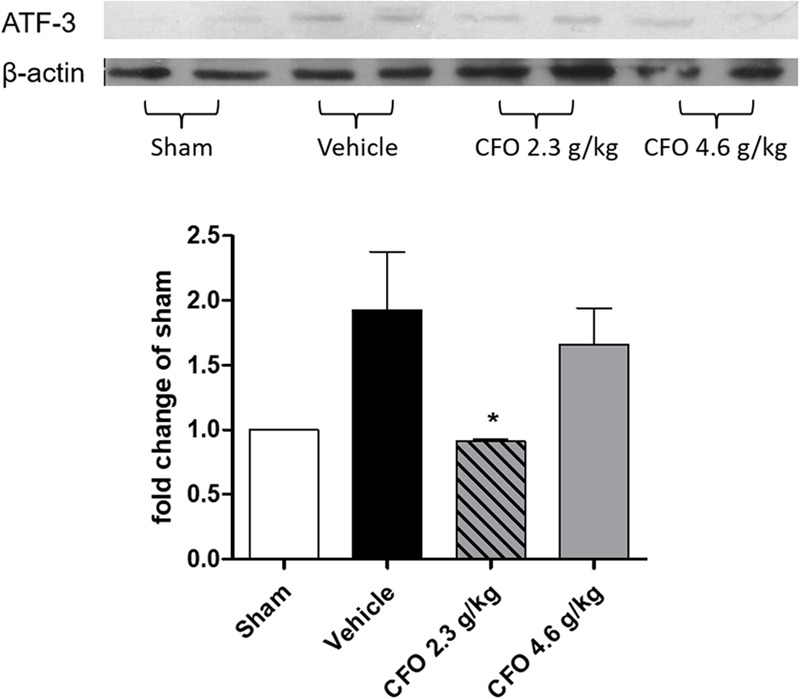
Concentrate fish oil treatment on ATF-3 expression after PSNL. Western blot analysis of neuronal damage marker ATF-3 in DRG at 9th day. *n* = 4; One-way ANOVA followed by Dunnet’s *post hoc* test, ^∗^*p* < 0.05 compared to vehicle group.

Activation transcription factor 3 (ATF-3) is a neuronal injury marker upregulated on DRG neurons after neuronal damage or stress (Tsujino et al., 2000). Quantification of ATF-3 on L-4 to L-6 DRGs ipsilateral to sciatic injury on the 9th day after PSNL showed that nerve injury induced a 2-fold increase compared to sham group, but in CFO 2.3 g/kg treated group neuronal damage was significantly reduced (**Figure 4**). Animals receiving CFO at 4.6 g/kg did not display a comparable reduction of ATF-3 expression.

### CFO Improved Motor Function and Nerve Regeneration

**FIGURE 5:**
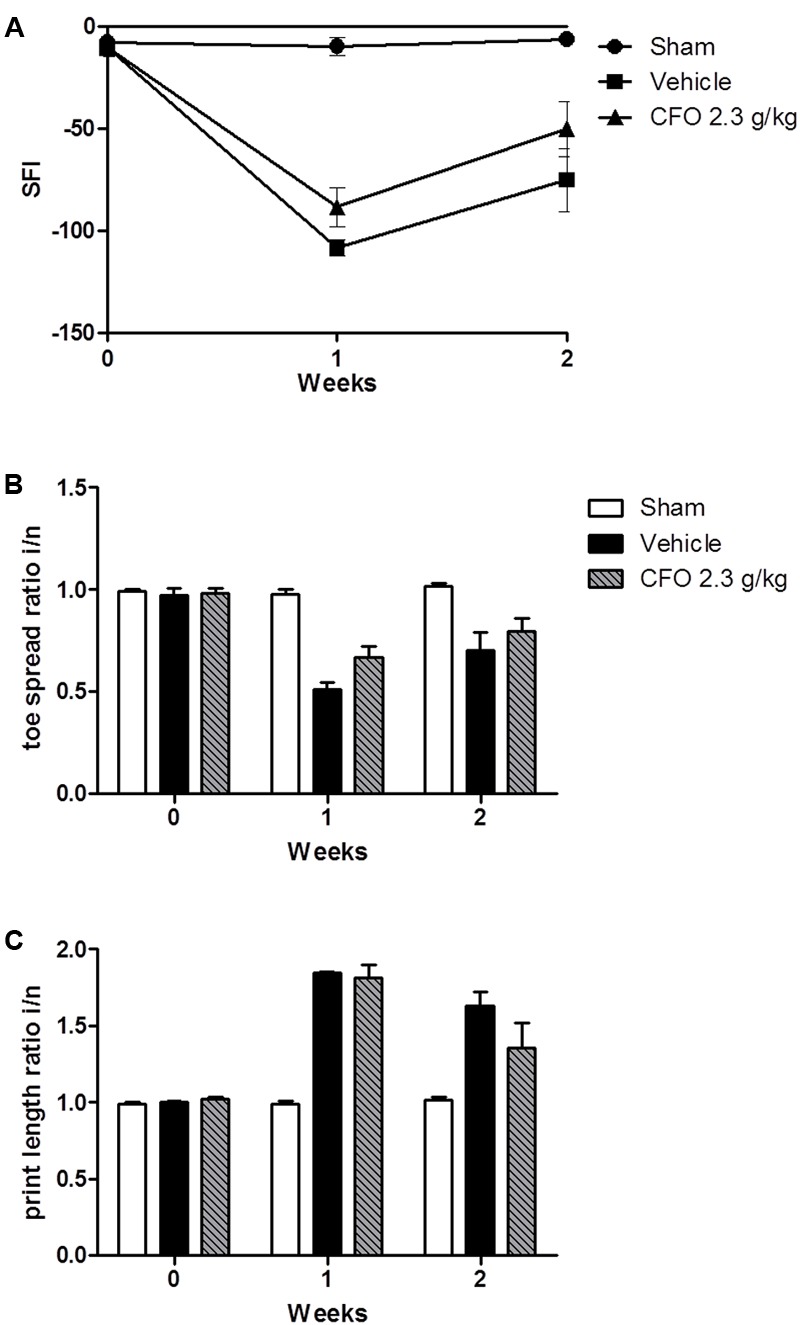
Effect of CFO on nerve function. SFI assessment after PSNL **(A)**, toe spread **(B)** and print length **(C)** ratios of the injured and normal paws at 1 and 2 weeks following sciatic ligation. *n* = 4–6; *t*-test compared to vehicle group.

Omega-3 PUFAs were effective in reducing inflammatory parameters, neuronal damage, and painful behavior in animals undergoing nerve injury. Thus, we asked whether CFO would have an effect on nerve regeneration and motor functional recovery. To evaluate sciatic nerve function, animals were treated for 2 weeks following injury and sciatic functional index (SFI) was calculated based on toe spread (TS) and print length (PL) measures of the injured and contralateral hind paws on weeks 1 and 2. One week after partial sciatic ligation an impaired nerve function was observed based on SFI, reaching the lowest value on a 0 to -100 scale in vehicle treated group in contrast to sham animals in which the index remained near 0 (**Figure 5A**). CFO 2.3 g/kg induced a modest increase in SFI in both weeks indicating that the combination of the ω-3 PUFAs, EPA and DHA, improves motor functional recovery (**Figure 5A**). The TS and PL ratios were also calculates separately (**Figures 5B,C**).

**FIGURE 6:**
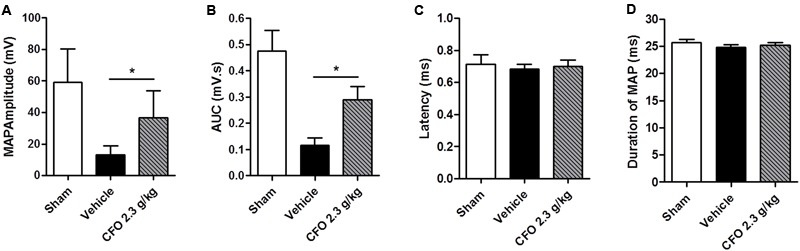
Electrophysiological recordings following sciatic nerve stimulation. Muscle action potentials (MAPs) were recorded 14 days after injury and treatment with CFO or vehicle. Amplitude **(A)**, AUC **(B)**, latency **(C)** and duration **(D)** were calculated from MAP. *n* = 7; *t*-test, ^∗^*p* < 0.05.

At the end of the second week of treatment, electrophysiological studies were conducted to assess sciatic nerve function. To that end, an electrical stimulus was applied proximal to the sciatic notch of anesthetized animals and recordings of the gastrocnemius MAPs were obtained. There was a substantial decrease on wave amplitude and area under the curve of MAPs of vehicle treated animals compared to sham operated animals. Conversely, CFO 2.3 g/kg treatment increased significantly both parameters compared to vehicle group (**Figures 6A,B**). The latency time and duration of recorded MAPs were not altered among the three groups evaluated (**Figures 6C,D**).

**FIGURE 7:**
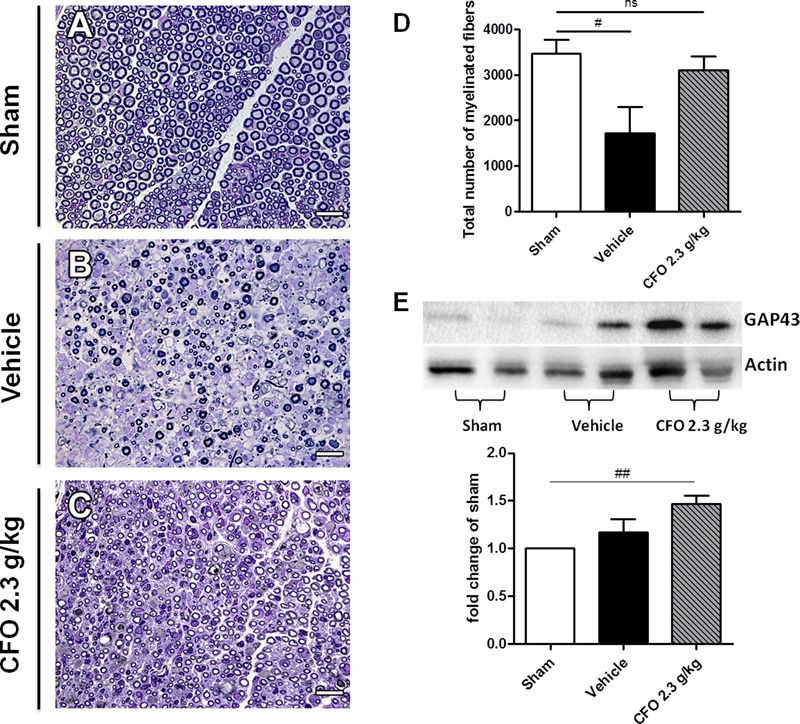
Regenerative effect of CFO on sciatic nerve. Histological analysis of sciatic nerve 14 days post-lesion. Light micrographs of semi-thin cross sections of the regenerating sciatic nerve. Sham **(A)**, Vehicle **(B)**, CFO 2.3 g/Kg **(C)** and quantification of the total number of myelinated fibers **(D)**. Scale bar: 20 μm **(A–C)**, *n* = 5–6 **(A–D)**. Expression of GAP-43 marker **(E)**, *n* = 4; One-way ANOVA followed by Bonferroni *post hoc* test, ^#^*p* < 0.05, ^##^*p* < 0.01 compared to sham.

Considering that the nerve function is influenced by myelin sheath integrity, the sciatic nerve of mice submitted to electrophysiological studies were collected and histological analysis of total myelinated fibers was performed (**Figures 7A–C**). Total count in sham group was approximately 3000 myelinated fibers; whereas nerve injury resulted in a decreased number of myelinated fibers to near 1400 on vehicle treated group. Supporting electrophysiological results, the mean of myelinated fibers on CFO supplemented mice was significantly elevated to about 3000 fibers at the level of sham animals (**Figures 7A,D**).

GAP-43 is a known marker to detect nerve regeneration. Therefore, GAP-43 expression in sciatic nerve 2 weeks after surgery was quantified in order to determine the regenerative properties of CFO. Although it was observed an increase in GAP-43 expression on both vehicle and CFO groups in comparison with sham group, only CFO treated animals presented a significant increment (**Figures 7B,E**). In addition, CFO group presented a tendency toward a higher expression of GAP-43 compared to vehicle group. Taken together, these results indicate that CFO seems to accelerate the regenerative process subsequent to a peripheral nerve injury, promoting a faster remyelination and a functional recovery.

### Established Neuropathic Pain Behavior Is Reverted by CFO Treatment

Since CFO was able to prevent mechanical allodynia and thermal hypernociception when given daily from the day of nerve damage, we decided to evaluate its potential to reverse established NP symptoms. Thus, mice undergoing PSNL were treated at the 5th day after surgery for 5 consecutive days with CFO 2.3 g/kg or 4.6 g/kg p.o.

**FIGURE 8:**
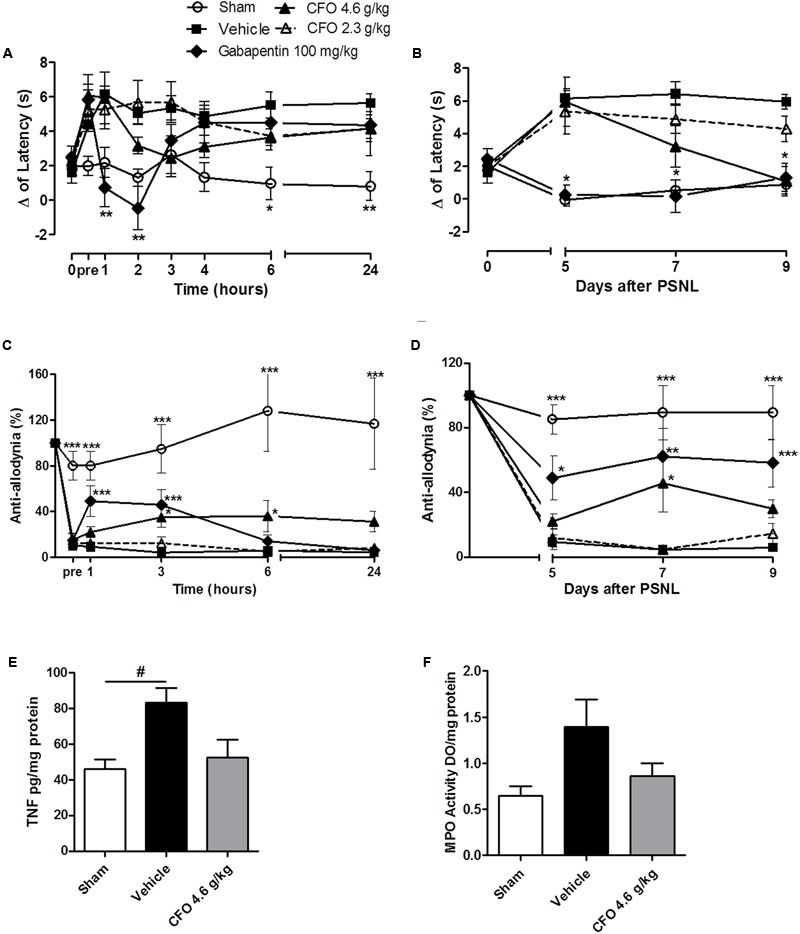
Concentrate fish oil effect on established neuropathic pain. A single daily oral administration of vehicle, CFO or gabapentin was initiated on the 5th day of lesion during 5 days. Thermal hypernociception **(A)** and mechanical allodynia **(C)** following treatment on 5th day after PSNL. Thermal **(B)** and mechanical **(D)** hypersensitivites on 5th, 7th, and 9th days 1 h after oral treatment. *n* = 5–6; Two-way ANOVA followed by Bonferroni *post hoc* test, ^∗^*p* < 0.05, ^∗∗^*p* < 0.01, ^∗∗∗^*p* < 0.001 compared to vehicle group. TNF level on spinal cord **(E)**, *n* = 4. MPO activity as indicative of neutrophils presence in sciatic nerve **(F)**, *n* = 3–4. One-way ANOVA followed by Bonferroni *post hoc* test, ^#^*p* < 0.05 compared to sham.

On thermal hypernociception, CFO 4.6 g/kg presented anti-hyperalgesic activity beginning on the 2nd hour of assessment on 5th day, although no statistical significant was observed. In contrast, gabapentin 100 mg/kg, a first line drug for NP, reverted thermal sensitivity for up to 2 h (**Figure 8A**). Interestingly, after repeated daily oral administration, CFO 4.6 g/kg on the 9th day completely abolished hypernociception (**Figure 8B**).

On the 5th day, mechanical allodynia was effectively reduced by CFO 4.6 g/kg 3 h following the first administration. This effect was sustained for up to 24 h opposed to Gabapentin 100 mg/kg treated group that exhibited anti-allodyinic effect for up to 3 h maximum (**Figure 8C**). On the following days mechanical allodynia was maintained partially reverted after CFO 4.6 g/kg oral administration (**Figure 8D**). No considerable efficacy was observed for CFO 2.3 g/kg on mechanical allodynia and thermal hypernociception at any time point.

In agreement with behavioral results, inflammatory parameters also seemed to be attenuated by CFO 4.6 g/kg on sciatic nerve and spinal cord. TNF levels in the spinal cord on the 9th day after nerve injury was reduced in 40% in mice treated with CFO (*p* = 0.51) (**Figure 8E**). Animals receiving CFO 4.6 g/kg also diminished MPO activity on sciatic nerve compared to vehicle mice (**Figure 8F**).

## Discussion

The present work demonstrates the effect of combined long-chain omega-3 fatty acids (CFO), EPA and DHA, in nerve regeneration and prevention of NP in mice, shedding light on its mechanism of action. Our results demonstrate that CFO is more effective in preventing NP related symptoms when administered soon after nerve injury. This effect is likely to be related to reduced neuroinflammation and neuronal damage as well as increased regenerative activity and motor recovery. It is noteworthy that CFO also decreased painful behavior in animals with well-established NP symptoms, indicating its preventive and therapeutic potential for peripheral NP.

Peripheral nerve injury as a result of disease or damage affecting somatossensory fibers is a frequent cause of NP leading to abnormal sensory processing (Colloca et al., 2017). PSNL is a well-established animal model of NP that elicits robust mechanical and thermal hypersensitivity that is correlated to human painful symptoms (Jaggi et al., 2011). In this study, 5 days after injury, animals presented consistent mechanical allodynia and thermal hypernociception observed in vehicle treated group. However, a daily dose of CFO at 2.3 g/kg was able of preventing reduction in mechanical withdrawal threshold associated with sciatic injury. A similar pattern was detected for thermal sensitivity. Interestingly, CFO also partially reverted mechanical allodynia without any significant alteration on thermal hypersensitivity.

Indeed, previous studies have reported analgesic properties of FO in thermal nociception and in mechanical allodynia associated with diabetic neuropathy (Redivo et al., 2016). DHA alone has also shown anti-nociceptive effect attenuating inflammatory pain, NP, and painful diabetic neuropathy in rodents (Lu et al., 2013; Manzhulo et al., 2015). Accordingly, the benefits from ω-3 PUFAs consumption in NP management has been reported in a study in which heterogeneous NP patients achieved significant pain alleviation with FO treatment (Ko et al., 2010).

Additionally, CFO has demonstrated a long-lasting effect without displaying any considerable side effects on locomotor activity. It has been shown that administration of FO in diabetic rats reduced mechanical allodynia and did not altered locomotion in the open field test (Lu et al., 2013). In contrast, gabapentin, which is a first line drug for NP therapy, presented anti-hypernociceptive effect only for up to 3 h and significantly reduced exploratory activity of animals. Actually, gabapentin is known for inducing somnolence and drowsiness at therapeutic doses besides presenting a short half-live (Dworkin et al., 2007). These findings suggest that CFO presents a safe and efficacious profile.

After nerve damage, immune cells support neuroinflammation through the release of several mediators including cytokine and chemokines (Scholz and Woolf, 2007). Initial cells migration, especially of neutrophils and macrophages, is a fundamental step which promotes peripheral sensitization and contributes to abnormal neuronal activity (Scholz and Woolf, 2007). Soon after injury, neutrophils are the first immune cells to migrate into the site of injury amplifying inflammatory response by recruiting macrophages and contributing to NP hypersensitivity (Perkins and Tracey, 2000). Regarding to inflammatory mediators, TNF, one of the major cytokines released during neuroinflammation, has been demonstrated to be essential for NP because of its peripheral and central actions driving hypersensitivity development after nerve injury (Hess et al., 2011; Park et al., 2011). In this context, our results showed that CFO decreased TNF levels in the spinal cord and reduced neutrophil migration, indirectly evaluated by MPO activity, in both protocols of treatment. A possible modulation of glial cells, which are considered the main producers of TNF at the spinal cord, may be involved in these effects (Ignatowski et al., 1999; Hanisch, 2002). Also, it was previously demonstrated that DHA modulates microglia activation through inhibition of TNF release in cultured cells and FO supplementation was able of decreasing TNF levels in rats DRGs (Antonietta Ajmone-Cat et al., 2012; Li et al., 2015). Considering neutrophil role in NP development and maintenance, it has been observed that depletion of these cells at the time of nerve injury attenuate hypersensitivity, however, the same effect is not detected after later depletion (Perkins and Tracey, 2000; Zuo et al., 2003). Therefore, considering the anti-inflammatory properties of EPA and DHA and the results obtained in this study, our data reinforces that CFO seems to modulate neuroinflammation in NP animals.

Neuroinflammation is one of the main drivers of neuronal abnormal activation after nerve injury. ATF-3 is a neuronal injury marker which expression is induced in DRG neurons rapidly after a nerve injury (Tsujino et al., 2000). Another important result emerging from this study is the reduced ATF-3 expression in DRGs of CFO 2.3 g/kg treated animals, indicating attenuation of neuronal damage. This finding is in accordance with a previous study conducted with fat-1 animals with elevated endogenous levels of ω-3 submitted to nerve crush model that displayed reduced ATF-3 expression in DRG (Gladman et al., 2012). Interestingly, ATF-3 seems to be not only related to injury, but also to a regenerative capacity of injured neurons since its expression is reported in neurons with successful regeneration and reinnervation (Obata et al., 2003; Seijffers et al., 2007).

Next, we asked whether CFO would be inducing a regenerative response contributing to the overall improvements observed. To answer that question, nerve function and regeneration were assessed by SFI and electrophysiological recordings in an extended treatment protocol for 14 days. In line with previous findings, CFO 2.3 g/kg improved nerve function evidenced by SFI and electrophysiological parameters after 1 and 2 weeks of treatment. These findings corroborate the improvement in SFI observed for fat-1 mice 1 week after being submitted to nerve crush injury (Gladman et al., 2012). Especially when accompanied by electrophysiological studies, the SFI presents a strong evidence of nerve injury recovery and regeneration (Navarro, 2016). In combination with mechanical and thermal nociceptive behavioral analysis, these results represent compelling evidence that oral supplementation of ω-3 PUFAs promotes reestablishment of sensory and motor functions after nerve ligation.

Nerve regeneration process comprises a series of events from neuroinflammation and Schwann cells activation to expression of key genes and myelin remodeling, strongly implicated in NP development (Wang et al., 2015). Our results showed that CFO treated mice presented a similar number of myelinated fibers to sham animals, opposed to the low count in vehicle treated animals. This result suggests nerve recovery at the tissue level. In addition, when the expression levels of GAP-43 were quantified, the group treated with ω-3 exhibited a marked increase compared to vehicle group, suggesting that CFO stimulates nerve regeneration. In contrast, GAP-43 expression was not induced in fat-1 mice, which may be explained by the ω-3 PUFAs profile in those animals predominantly composed by an increment in DHA and DPA in the spinal cord and DRG, but unaltered EPA levels (Gladman et al., 2012).

The indication of regeneration observed in sciatic nerve partially explains the results found in this study since nerve regeneration is a long process and our results were obtained in a short window of 2 weeks, which suggests that CFO could also present neuroprotective effect. Further investigation is required to clarify if the regenerative potential of CFO is due to neuroprotective mechanisms, Moreover, approaches to improve neural tissue repair locally, such as introduce ω-3 PUFAs into nerve conduits implants, could arise as useful strategy to investigate ω-3 PUFAs local effects (Yin et al., 2012; Mu et al., 2014). The exact role of ω-3 PUFAs in many neurological conditions is also uncertain. A growing number of studies have explored the neuroprotective effect of ω-3 PUFAs, notably DHA, in spinal cord injury (SCI), Alzheimer’s disease and retinal ganglion cells injury model (Hashimoto et al., 2002; Lim et al., 2013; Peng et al., 2016), whereas others have pointed to the regenerative properties of ω-3 PUFAs in SCI and peripheral nerve injury (Gladman et al., 2012; Liu et al., 2015). ω-3 PUFAs possess many biological functions and are essential for the development of the nervous system. Because endogenous synthesis cannot occur, ω-3 PUFAs integrate the class of essential FAs obtained through diet (Dyall and Michael-Titus, 2008; Calder and Yaqoob, 2009). DHA is the most abundant PUFA in cell membrane composition and is the main studied ω-3 as modulator of the nervous systems (Bazan, 2007). Regarding to pain, it is described for DHA an antinociceptive effect after thermal and chemical stimuli and also prevention of NP development through the modulation of macrophage and microglia (Nakamoto et al., 2010; Manzhulo et al., 2015). Also, the exact mechanisms involved in these effects are inconclusive with reports showing that ω-3 PUFAs could activate GPR120 signaling, modulate TRPV1, and interfere with cytokines gene transcription via NF- κB and PPARγ (Matta et al., 2007; Oh et al., 2010; Calder, 2013).

Even though DHA and EPA are proven to directly modulate neuronal functions, the hypothesis of endogenous conversion of these ω-3 PUFAs into other mediators contributing to the outcomes observed is plausible. Anti-inflammatory and pro-resolving lipid mediators such as eletrophilic fatty acid oxo-derivatives (EFOX) and resolvins series E and D deriving from EPA and DHA, respectively, have been studied due to their neuromodulatory and anti-inflammatory effects (Ji et al., 2011; Dyall, 2015). Our group previously published that orally administered ω-3 increased tissue levels of resolvin D1 in rats after inflammatory stimuli (Lobo et al., 2016). Thus, it is possible that our results reflect, at least partially, the effects of endogenously derived lipid mediators. Also, as in the composition of the CFO used in this study is present alpha-tocopherol as anti-oxidant, the possibility of its contribution to the observed effects cannot be excluded without further investigation as well.

The exact mechanisms by which the combination of EPA and DHA exerts its preventive effect on peripheral nerve injury remain to be elucidated and its potential neuroprotective effect is to be investigated. However, our results points to a combination of multiple targets involving both neuronal and immune components leading to anti-inflammatory, anti-nociceptive and regenerative effects. Our behavioral findings are new to the extent that prevention of NP development and indication of sensorial and motor recovery were obtained with ω-3 PUFAs, emphasizing their prophylactic and therapeutic potential in peripheral nerve injury.

## Conclusion

The data presented in this study suggest that CFO, a combination of the long chain ω-3 PUFAs EPA and DHA alleviates pain hypersensitivity and promotes functional nerve recovery presenting a safe and efficacious alternative for prevention and treatment of peripheral nerve injury-induced NP.

## Author Contributions

All authors listed have made a substantial, direct and intellectual contribution to the work, and approved it for publication. JO and AMBM designed, supervised, analyzed, and provided reagents to the electrophysiological, microscopic and sciatic functional index studies. Regarding the *in vivo* and *in vitro* data RS, CL, JO, BS, and FD conducted experiments, acquired and analyzed data. CL, RS, and ALPM designed research studies, analyzed data and provided reagents. RS, CL, and ALPM wrote the paper. ALPM, CL, JO, and AMBM revised the paper critically for important intellectual content.

## Conflict of Interest Statement

The authors declare that the research was conducted in the absence of any commercial or financial relationships that could be construed as a potential conflict of interest.
